# Human-AI Interaction in Kidney Transplant Decision Support Systems: Qualitative Study of Patient and Support Person Expectations

**DOI:** 10.2196/83195

**Published:** 2026-05-21

**Authors:** Zeineb Sassi, Sascha Eickmann, Roland Roller, Bilgin Osmanodja, Jakob Joachim Spencker, Ömer Ege Ömeroğlu, Aljoscha Burchardt, Michael Hahn, Peter Dabrock, Sebastian Möller, Klemens Budde, Anne Herrmann

**Affiliations:** 1 Department of Epidemiology and Preventive Medicine, Medical Sociology, Faculty of Medicine, University of Regensburg, Dr. Gessler-Straße 17, Regensburg, 93051, Germany, 49 17677254997; 2 Central Clinical School, University of Sydney, Sydney, NSW, Australia; 3 Institute of General Practice, University Hospital Regensburg, Regensburg, Bavaria, Germany; 4 German Research Center for Artificial Intelligence (DFKI), Berlin, Germany; 5 Department of Nephrology and Medical Intensive Care, Charité-Universitätsmedizin Berlin, Corporate Member of Free University of Berlin, Berlin Institute of Health, Berlin, Germany; 6 Department of Nephrology and Medical Intensive Care, Charité-Universitätsmedizin Berlin, Corporate Member of Free University of Berlin, Berlin Institute of Health, Humboldt-University of Berlin, Berlin, Germany; 7 Chair for Systematic Theology II (Ethics), Friedrich-Alexander-Universität Erlangen-Nürnberg (FAU), Nuremberg, Germany; 8 Quality and Usability Lab, Technical University of Berlin, Berlin, Germany; 9 Department of Internal Medicine III, University Hospital Regensburg, Regensburg, Germany; 10 Bavarian Centre for Cancer Research (BZKF), Regensburg, Germany

**Keywords:** artificial intelligence, AI, decision-making, medical informatics, decision support system, DSS, health care, human-computer interaction

## Abstract

**Background:**

Artificial intelligence (AI) is increasingly applied in medicine, including clinical decision-making. AI-based decision support systems (DSS) can enhance early risk detection and treatment optimization. However, the perspectives of patients and their support persons on AI-assisted DSS in clinical care, particularly regarding shared decision-making (SDM), remain underexplored.

**Objective:**

This study investigates the expectations, informational needs, and perceptions of patients who underwent kidney transplantation and their support persons regarding AI-assisted DSS and its influence on SDM in posttransplant care.

**Methods:**

In a longitudinal qualitative study, 36 semistructured interviews were conducted with patients who underwent kidney transplantation and their support persons at a German kidney transplant center. Participants were asked about their views on AI’s role in follow-up care, its impact on communication, trust, and decision-making, as well as their informational needs regarding AI-assisted DSS. Interviews were transcribed, pseudonymized, and analyzed using framework analysis.

**Results:**

Participants recognized AI’s potential to support clinicians by identifying risks of transplant loss, rejection, and infection, and by providing data-driven treatment recommendations. However, they emphasized that final decisions should remain with physicians. A majority of participants (n=28, 78%) expressed concern that AI might depersonalize care and diminish physician-patient communication due to a lack of “human touch.” Participants demonstrated limited understanding of AI-based DSS functionality and highlighted the need for simple, accessible educational materials (eg, leaflets) explaining AI operations. While most doubted AI could replicate human empathy, some acknowledged that AI might be perceived as more attentive than time-pressured physicians, offering consistent monitoring and support. Participants consistently stressed that AI should augment, not replace, clinical decision-making.

**Conclusions:**

Patients who underwent kidney transplantation and support persons endorse the integration of AI in follow-up care when it enhances clinical decision-making without supplanting the physician’s role. Acceptance and trust depend on transparency, accountability, and preserving the “human touch” in care. The development of educational tools to communicate AI functions and limitations is crucial to empower patients and support persons in SDM processes and to ensure AI complements, rather than undermines, patient-centered care.

## Introduction

### Impact of AI on Medical Decision Support in Nephrology

Artificial intelligence (AI) has the potential to transform health care by improving diagnostic accuracy, supporting medical decision-making, and enhancing patient care [1]. In various medical fields such as radiology, oncology, and cardiology [2], the successful use of AI has supported early detection, personalized treatment planning, and risk prediction, providing a comparative context for its emerging role in nephrology, where AI-based decision support systems (DSS) are growing as valuable tools to assist health care professionals. In the context of posttransplant care, particularly for patients who have undergone kidney transplantation, these systems may predict outcomes such as graft survival, detect patients at risk for rejection, and optimize medication regimens. They can improve patient management by identifying trends and patterns in patient data that may not be easily recognized by human physicians [3]. Posttransplant care involves complex, long-term decision-making processes that require close monitoring and individualized treatment adjustments. For example, managing immunosuppressive therapy demands high levels of patient-physician interaction, making kidney transplant recipients particularly sensitive to changes introduced by AI-based DSS [1-4]. By analyzing extensive patient data, AI systems provide insights—such as predictive modeling for graft survival—that complement traditional clinical assessments [5]. While AI offers significant potential to enhance posttransplant care, integrating these technologies into clinical practice presents several challenges. Effective AI systems depend on high-quality, standardized data. However, health care data often originates from diverse sources with varying formats and standards, leading to interoperability and integration challenges [3,6,7]. Such a lack of standardization impedes AI model training and deployment, potentially compromising their accuracy and reliability [8]. Moy et al’s [9] scoping review highlights that patients’ needs and expectations are often overlooked in the development and application of AI technologies, underscoring the critical need to incorporate patient and end-user perspectives in health care AI design [10]. Patients are rarely involved in the design of AI systems and often have a limited understanding of how these technologies work, including their underlying data and limitations. This lack of knowledge may foster uncertainty and unaddressed concerns, which in turn can undermine trust [9,10]. To counteract this, it is essential to identify and integrate the expectations and needs of patients and their support persons, as their preferences directly shape the acceptance and effectiveness of AI technologies in health care.

### Shared Decision-Making: The Importance of Understanding the Preferences and Concerns of Patients in Medical Decisions

Shared decision-making (SDM) refers to a collaborative process in which patients and physicians jointly make treatment decisions, with patient values and preferences serving as a central guiding principle [11]. In the context of AI, SDM gains particular importance, since patients often hold complex and ambivalent views on the role of AI in their care [12]. In post–kidney transplant follow-up care, patient engagement in SDM is vital, as treatment decisions often involve balancing complex risks, such as graft rejection [13,14]. Patients fear that AI may reduce the “human touch” of health care, leading to depersonalized treatment [15,16]. They also express worries about data privacy and security [17,18], potential diagnostic errors or misjudgments in rapidly changing health conditions [18-20], and a lack of transparency in algorithmic decision-making [20,21]. Such concerns highlight the need to understand patient perspectives and integrate them into SDM, as acceptance of AI-based decision support depends on patients’ trust and willingness to engage with these technologies.

While prior literature has addressed general patient attitudes toward AI in health care, empirical evidence on the expectations of patients and support persons regarding AI-based SDM in nephrology remains lacking. Most studies focus on physician perspectives, leaving the voices of patients and support persons under-represented [9]. In Germany, there is no qualitative evidence on experiences with AI-based care in high-stakes contexts such as kidney transplantation. This study addresses this gap by providing first insights into expectations and needs related to AI-based DSS within a German transplant center.

### Support Persons’ Role in AI-Assisted SDM Matters

Support persons, such as family members, are key sources of patients’ health information and play vital roles in diagnosis, treatment, and recovery [22]. Previous research indicates that surrogate decision-makers, such as family members, make approximately 75% of the decisions for hospitalized patients with life-threatening illnesses [23]. Despite the importance of support persons in patient care, their perspectives—particularly in posttransplant contexts—remain largely underexplored [24,25]. Support persons also face unique concerns when participating in decision-making throughout a patient’s health care journey, with these concerns being intensified by the introduction of AI. Navigating complex medical AI systems adds both emotional and practical challenges, as support persons must assist patients while also understanding and interpreting AI-generated data and recommendations [4]. One prominent concern for support persons is information overload, where frequent AI alerts and large volumes of data can create cognitive stress rather than provide support. Support persons also worry that AI may weaken the emotional connection between patients and their treating physician, potentially leading to feelings of isolation [26,27]. Additionally, support persons fear that over-reliance on AI may reduce personalized care in medical decision-making [28], and they may feel uncertain about endorsing treatment decisions if they do not understand how AI functions or how it influences patient care [29].

In all, these emotional and practical concerns illustrate that support persons are not only critical to patient care but also pivotal stakeholders in AI-based SDM. Understanding and integrating their perspectives and expectations are, therefore, essential to ensure that AI systems support both patients and their support persons effectively.

### Patient-Centered Care Through Studying the Perspectives of Patients and Support Persons on AI-Based Decision-Making

AI-based DSS are increasingly applied in high-stakes medical contexts, including posttransplant care, where they can support complex clinical decisions. The effectiveness of these systems, however, depends not only on technical performance but also on their alignment with patient-centered care principles, particularly SDM, which integrates patient preferences, values, and the perspectives of support persons [11,23,30].

Patients and support persons bring their own expectations and concerns to medical decision-making, which strongly influence the acceptance of AI. Previous research indicates that mistrust in medical AI is common and often stems from limited knowledge of its functioning, personal experiences, and concerns about data privacy, potential errors, and the depersonalization of care [31]. Support persons, who frequently act as surrogate decision-makers and key sources of health information for patients [32], face additional challenges, including the cognitive burden of AI-generated information, uncertainty about AI’s impact on patient outcomes, and anxiety that AI may reduce personalized care [4,24,26-29,32]. Emotions such as anxiety play a decisive role in shaping support persons’ engagement with AI-based technologies, influencing whether they accept or reject these tools [32].

Despite these insights, qualitative research examining how AI-based DSS affects SDM in nephrology remains scarce. Existing studies often focus on general patient attitudes or physician perspectives, leaving the voices of patients and support persons under-represented. This study addresses this gap by exploring the expectations and needs of patients and support persons regarding AI-based DSS in posttransplant care. By capturing these perspectives, the research aims to inform the development and implementation of AI technologies that support SDM, enhance patient autonomy, foster trust, and align with patient-centered care principles, such as transparency and accountability.

## Methods

### Study Design

This study is part of a 2-year longitudinal qualitative interview study conducted as part of an interventional study in a German kidney transplant center (KTC). The protocols for both the interventional study as well as the nested interview study were published before data collection, and the study has been registered (NCT06056518) [13,14]. Semistructured interviews with patients who underwent kidney transplantation and their support persons were conducted at baseline and are reported here. The AI-based DSS applied in this study—a predictive machine learning model—assesses the risk of graft loss within the upcoming 360 days for patients who underwent kidney transplantation [28,33]. The system was rigorously developed and pretested before deployment and is monitored by a team of technicians supporting the technical subproject of this study [28,34].

### Sample and Recruitment

Eligible patients were identified by study physicians after applying all inclusion and exclusion criteria to the entire transplant cohort of the KTC. Ongoing analysis revealed thematic redundancy in later interviews, indicating sufficient thematic depth and suggesting that data saturation had been reached.

### Inclusion and Exclusion Criteria

Patients were eligible if they were 18 years or older, had undergone kidney transplantation, and had a functioning kidney graft with an estimated glomerular filtration rate of less than 30 mL/min/1.73 m^2^. They were scheduled for routine follow-up at the participating KTC and provided written informed consent. Patients and their support persons were able to communicate in German. Support persons were eligible if they were 18 years or older and could provide informed consent.

### Data Collection

Consenting patients and support persons were contacted by a researcher to arrange an interview appointment. Participants could choose the interview mode according to their preferences. All participants indicated that they preferred to be interviewed via telephone. Interviews were scheduled according to the availability of the patients and support persons to reduce research-related burden on participants. Through semistructured interviews, participants were encouraged to express their views on how AI-based DSS may impact physician-patient–support person communication and the decision-making process in the way they preferred. A narrative approach was used to elicit the variety and interplay of potential factors related to physician-patient communication in this area. This open approach naturally led to a relatively generic interpretation and assessment of AI. At the end of the interview, participants were given the opportunity to provide additional comments or ask questions. The researchers received intensive training from an interdisciplinary expert team in conducting these interviews to reduce bias in question framing, administration, and interpretation. Additionally, standardized interview protocols were used to document each interview.

### Interviews

The interview guide was developed based on a literature review [35,36] and discussions among the interdisciplinary research team, which included experts in medicine, communication and behavioral science, health services research, ethics, and computer science. Participants were asked about their expectations regarding the use of AI-based DSS, particularly in the context of SDM. Topics included patients’ communication preferences, perceptions of trust, transparency, and responsibility in relation to AI-based DSS, as well as anticipated benefits, risks, and barriers to implementing this tool in routine care [14]. The interviews were conducted by a researcher with a background in psychology and health services research, along with prior academic engagement with topics related to AI in health care, which informed a sensitized but critically reflective stance toward AI throughout data collection and analysis. This disciplinary perspective may have influenced the framing of questions and participants’ reflections on digital tools. Regular reflexive discussions within the interdisciplinary research team were used to critically examine potential assumptions during data collection.

### Data Analysis

Interviews were transcribed verbatim. Two trained researchers reviewed each transcript for accuracy by comparing it with the original audio recordings, correcting any transcription errors, and ensuring completeness.

The data were then analyzed using framework analysis, which allows for both inductive and deductive coding [37]. Initially, 2 researchers conducted independent open coding, identifying concepts and patterns emerging directly from the data (inductive coding). Based on these initial insights and the study’s guiding research questions, a framework of analytical categories was developed and applied to the data (deductive coding) [37]. The deductive coding framework was informed by established SDM [11,30,38,39] and human-computer interaction models [40], which guided the development of analytical categories related to trust, transparency, responsibility, and collaborative deliberation. In addition, principles from human-centered AI [39] and the European Commission’s Ethics Guidelines for Trustworthy AI [41] informed categories concerning explicability, accountability, and stakeholder engagement.

To enhance the credibility and consistency of the coding process, 2 researchers discussed discrepancies and reached a consensus through iterative refinement of the coding framework. The qualitative data management software, ATLAS.ti (ATLAS.ti GmbH), was used to organize, code, and retrieve relevant data segments efficiently. The results of the analysis were discussed in close collaboration with the entire interdisciplinary research team.

### Ethical Considerations

All patients were provided with written informed consent for the study intervention as well as the interview study. The support person accompanying the patient to the appointment was also provided with written informed consent [14]. The ethics committee of Charité - Universitätsmedizin Berlin approved this study (EA1/177/23; date of approval: September 19, 2023). Data were pseudonymized, and the confidentiality and privacy of participants were strictly maintained in accordance with institutional and national ethical standards and the Declaration of Helsinki. Each participant received financial compensation of €50 (approximately US $58.60).

## Results

### Overview

A total of 36 participants were included in the final analysis. Of these, 22 (61%) were males and 14 (39%) were females. Interviews were conducted together with a support person in 21 (58%) cases; among those, 9 (43%) support persons were identified as the participant’s spouse. Fifteen (42%) patients stated that they preferred not to involve a support person in the call. Participants’ ages ranged from 37 to 74 years (mean 57, SD 11 y). Sixteen participants resided in Berlin, whereas 20 lived elsewhere in Germany (Table 1). The duration of the interviews varied, with an average length of 34 (SD 14) minutes. The median interview length was 32 (IQR 28-42; range 20-90) minutes. Main themes derived from the Interview data are summarized in Table 2. Patients’ sociodemographic and transplantation-related characteristics are presented in Table 1 below.

**Table 1.** Patients’ sociodemographic and transplantation-related characteristics (N=36).

Characteristics	Values
Age (y), mean (SD; range)	57 (11; 37‐74)
Gender, n (%)	
Male	22 (61)
Female	14 (39)
Involvement of support person, n (%)	
Yes	21 (58)
Spouse	9 (43)
Other	12 (57)
No	15 (42)
Time since kidney transplantation (y), n (%)	
<1	0 (0)
1‐5	4 (11)
5‐10	6 (17)
>10	26 (72)
Place of residence, n (%)	
Berlin	16 (44)
Germany, not Berlin	20 (56)

### Patients’ Perceived Benefits and Expectations Regarding the Use of AI-Based DSS

Patients expressed mixed feelings when asked what they generally associate with AI in health care. Some (15/36, 42%) were open and optimistic, viewing AI as a helpful tool that could support them and their physicians by providing additional insights for individualized treatment and improved SDM. A commonly mentioned benefit was AI’s ability to process vast amounts of data quickly and potentially identify treatment options or risks that a human physician might overlook. As 1 participant explained:

*It can be a help, like if it reminds the doctor that a certain value is off and that medication B shouldn’t be given. That’s a help. But in the end, if it still gets prescribed, well, then it’s a human error.*

[Male, 57 years]

Patients also recognized the potential of AI to enhance communication between health care providers by transferring patient data, which could enable more coordinated and informed care.

Another participant emphasized AI’s potential to broaden clinical perspectives, stating that it may “show people possibilities that might not have come to their minds at that moment*”* (male, 55 years).

Importantly, participants emphasized that they expect AI not to make decisions independently.

“Yes, together with the doctor, yes—on its own, no” (male, 71 years), summarized a patient when asked about AI making medical decisions regarding his treatment. Another participant commented*,* “Definitely to be part of the decision-making—that’s exactly the trend that’s developing right now” (male, 63 years)*,* underscoring the growing desire for collaborative approaches in which AI supports, but, according to most participants, does not replace human judgment and patient involvement.

While many acknowledge that AI can provide rapid and comprehensive analysis, potentially identifying patterns in laboratory results or drawing insights from large datasets, participants emphasized that AI should only serve as a supportive tool in the decision-making process. As 1 patient with her support person put it:

*Ultimately, the doctor should still be the one in charge of my treatment decision. The AI should just support them. That’s my view.*

[Female, 50 years]

Another participant praised AI’s analytical capabilities but highlighted the importance of human oversight:

*The AI has much faster access to a lot more data than my treating physician. [...] It might raise a red flag when something is off, but then the doctor looks at it and says, “Okay, what’s going on here?” [...] And then we go back to the doctor-patient conversation, trust, and competence. That’s [the] key.*

[Male, 65 years]

These expectations closely align with established models of SDM: trust and transparency are core prerequisites for meaningful patient participation in SDM [11], particularly in complex and high-stakes contexts such as kidney transplantation. Participants’ insistence that AI should support rather than replace physicians reflects a preference for AI as an enabling tool within SDM, rather than as an autonomous decision-maker. Interpreting these findings through an SDM lens highlights that the value of AI-based DSS lies in strengthening, rather than disrupting, relational and communicative aspects of care.

### Patients’ and Support Persons’ Fears and Concerns Regarding AI-Based DSS

Most patients (26/36, 78%) expressed concerns about AI’s inability to replicate the human qualities of empathy and individualized care—care that meets their unique needs, preferences, and values to understand their decision preferences. They feared that AI-based care, including DSS, might create distance between them and their treating physicians, who, according to the patients, are better equipped to understand their concerns, unique circumstances, medical history, and preferences, thereby avoiding a one-size-fits-all approach. They emphasized that while AI could be helpful, physicians should not rely on “machines” and thus should not leave the decision solely to the AI-based DSS. The majority of patients and their support persons (30/36, 83%) emphasized that their trust in final decisions regarding their care should always rest with their treating physicians. Physicians should never rely too heavily on automated systems, as 1 patient noted:

[...]*It should never end up in a situation where the doctor no longer thinks and it becomes an automatic process, and if we were to be very dystopian, that eventually no doctor would be there at all, but rather we would feed data to the AI, which then says, "Do this and that," and we would only execute it [...] So, it [the DSS] should never replace a doctor!*

[Male, 57 years]

In addition to that, patients and their support persons also identified several weaknesses and potential fears in relation to AI-based DSS. The most common concerns were related to the lack of “human touch” (29/36, 80%), referring to the absence of empathy, emotional understanding, and personalized communication that physicians offer while navigating complex treatment decisions:

*Because human interaction still needs to be there [...] People don’t want to only talk to a computer. They also want to have the human element.*

[Male, 48 years]

Participants also expressed concerns about the potential for AI to make errors or miss important contextual information about a patient’s condition. Many patients feared that over-reliance on AI could lead to a loss of personalized care (31/36, 78%) and treatment decisions, where their unique needs and circumstances might be overlooked.

### Impact of Patients’ and Support Persons’ Knowledge Gaps on AI-Based DSS

Notably, many patients and support persons expressed a limited understanding of how AI-based DSS functions within medical settings (31/36, 86%), which often led to skepticism and uncertainty. Limited AI literacy emerged as one of the core findings in this study. A poor understanding of how AI-based DSS functions directly shaped participants’ levels of trust, acceptance, and their willingness to engage in AI-assisted SDM. In addition to that, uncertainty about data sources, decision logic, and error handling amplified concerns about over-reliance on AI and loss of control. These findings demonstrate that improving AI literacy is not merely an educational “nice-to-have,” but a prerequisite for meaningful and equitable AI-assisted SDM.

Another often-recurring theme was the concern about data privacy and the potential misuse of sensitive health information. Several participants voiced fears about who has access to their medical data and how it is processed, particularly if AI systems are used without sufficient transparency or oversight.

One participant reflected on this concern, saying, “If data about your body, your health, your care is stored and evaluated, it’s fine—as long as it’s not misused, and that’s my fear when I say this” (female, 72 years).

Another patient noted:

*I hope [AI] turns out to be positive. But it could also go the other way. After everything we’ve experienced in recent years, I’m not sure whether I could really put my trust in it. [...] Ultimately, the final decision should always be ours [Patient, SP and physician].*

[Female, 38 years]

This quote captures the cautious optimism expressed by many—acknowledging potential benefits while remaining wary of unintended consequences or over-reliance on technology.

Moreover, patients consistently emphasize the importance of transparent and accessible information about how AI is used in their posttransplant care. They expressed a desire to better understand the technology’s role, benefits, and risks to feel more secure and involved in decision-making processes. Transparency, for many, was a key factor in building trust in AI-assisted SDM.

Overall, while patients recognized AI’s potential to contribute valuable insights, many stressed that only a physician can contextualize these insights and make appropriate, individualized decisions through direct conversation and shared understanding. According to most of the participants, trust in AI, therefore, depends not just on its accuracy but also on how clearly its role is communicated and how responsibly it is integrated into the decision-making process.

### Perceived Impact of AI-Based DSS on SDM

Across interviews, participants expressed a strong desire to actively participate in the decision-making process between physicians and the AI-based DSS. Rather than seeing AI as a substitute for the physician or the patient’s perspective, many envisioned a triadic model of decision-making involving the patient and the support person as one unit, the physician, and the AI-based DSS—not as a third independent decision-maker, but as a supportive participant. This approach was perceived as enriching the process, increasing trust, and reinforcing the role of patients in their care.

One patient articulated this ideal of SDM powerfully:

*[...] But if the results are reviewed together and I, as the patient with my partner, can also help decide—then there are 3 things that can be brought into it: the technology, the doctor, and myself as the patient.*

[Male, 57 years]

Across multiple interviews, patients and their support persons consistently emphasized the importance of transparency in AI-based decision-making. They expressed a desire to understand not only how the AI system itself generates recommendations, but also how these recommendations are integrated into their physician’s decision-making process. For instance, one support person described an ideal scenario in which AI would become part of a joint consultation, allowing both the patient and the physician to review and discuss the AI’s input together:

*We [the patient and I] would find it good if we could look at the AI together with the doctor, for example, to see what data it has about him. If I had the chance to look at the computer, too. I would like that—that both the doctor and I see the same thing. So really a kind of teamwork with the AI, the doctor, and us.*

[Male, 63 years]

At the same time, not all participants felt sufficiently included in current decision-making processes. Some reported a lack of involvement and insufficient dialogue about treatment choices—particularly in relation to potential AI-based recommendations:

*If I were included, I would want to be asked: Did you understand this? Is it okay for you? But I don’t think I will be asked that.*

[Female, 38 years]

In summary, AI was seen as having the potential to enhance SDM, especially when its use facilitates dialogue, offers additional perspectives, or helps physicians explain and justify their decisions. However, participants made it clear that realizing this potential depends on the physician’s communication style and the extent to which the patient has been introduced to and informed about the AI and whether the patient is treated as an equal partner in the decision-making process.

It is important to note that participants’ perceptions varied depending on the type of AI system envisioned. References to predictive models were often associated with background data processing and risk estimation, whereas conversational or interactive AI was more frequently linked to concerns about authority, communication, and the involvement of patients.

The main themes and subthemes derived from the interview analysis are summarized in Table 2

**Table 2.** Main themes derived from interview data (N=36).

Theme	Values, n (%)
Previous experience with AI^a^-based medical devices	
Yes	21 (58)
Male	19 (90)
Female	2 (10)
No	15 (42)
General attitude toward the inclusion of AI in health care	
Rather optimistic	15 (42)
Rather pessimistic	5 (14)
Mixed	16 (44)
Trust in AI-based DSS^b^ regarding final treatment decisions	
More trust in AI-based DSS	0 (0)
More trust in treating physician	30 (83)
Mixed	6 (17)
Concerns expressed with the integration of AI in the treatment	
Perceived personal lack of knowledge about the AI-assisted DSS	31 (86)
Perceived lack of “human touch” (eg, empathy) of treatment	29 (80)
Fear of less personalized treatment	28 (78)
Other	16 (44)
Perceived impact of AI on SDM^c^	
Desire to include AI-assisted DSS in SDM	22 (62)
Desire for more transparency on how the AI-based DSS generates treatment recommendations	31 (86)
No perceived impact	0

a AI: artificial intelligence.

b DSS: decision support system.

c SDM: shared decision-making.

## Discussion

### Principal Findings

This qualitative study provides insights into how patients who have undergone kidney transplants and their support persons perceive AI-based DSS in their posttransplant care, especially in the context of SDM. While participants largely welcomed AI as a supportive tool, they consistently emphasized the irreplaceable role of human judgment and the fear of the loss of the “human touch,” meaning emotional connection and individualized care. These concerns appeared to have different origins. Some were grounded in participants’ prior experiences with AI technologies, such as impersonal digital systems or fragmented communication, which informed fears of further depersonalization. Other concerns reflected more general uncertainty toward AI as an emerging and poorly understood technology, particularly in relation to accountability and error potential. Distinguishing between experiential and abstract sources of concern helps to explain the breadth of apprehension expressed and highlights different points of intervention. The modality-specific perceptions were reflected in the empirical material, where participants associated predictive systems with passive support, while interactive AI was more often perceived as actively shaping communication and decision-making processes.

These findings point to a critical challenge for future implementation: how to meaningfully integrate AI into the clinical encounter without eroding the essential elements of SDM: Charles et al [42] define SDM as an interactive process requiring at least 2 active participants, typically the physician and the patient, each contributing to the decision with their expertise and values. As previously mentioned, in our findings, patients envisioned a triadic model involving the patient, the support person, and the treating physicians, in which AI serves not as a third independent decision-maker but as a supportive participant, providing additional information without replacing human actors. This aligns with Elwyn’s [11] model of SDM, which underscores the importance of team talk, option talk, and decision talk, emphasizing collaborative deliberation based on trust and clarity. However, our participants made it clear that this process depends heavily on whether AI can be transparently explained and meaningfully integrated into conversations, without diminishing their own agency or emotional safety.

### Patient- and Human-Centered AI: Empowering Patients Through Co-Design

Many patients lack an understanding of how AI-based DSS work, revealing a clear need for patient-focused approaches to AI development. Patient-centered and human-centered AI aim to ensure that these technologies are designed not merely for efficiency, but to align with patients’ values, expectations, and experiences. Co-design is crucial in this context: involving patients as partners in the development process helps ensure that AI systems are understandable, transparent, and sensitive to patients’ concerns [39]. International efforts, such as the European Commission’s Ethics Guidelines for Trustworthy AI, stress that AI must be explicable, human-centered, and designed with stakeholder engagement, foremost including patients. In addition, there is a growing recognition that explainable AI must be designed not only for regulatory transparency but also for patient comprehension [43]. While initiatives such as the SPIRIT-AI (Standard Protocol Items: Recommendations for Interventional Trials—Artificial Intelligence) guidelines [44] or the CONSORT-AI (Consolidated Standards of Reporting Trials—Artificial Intelligence) [45] extensions have focused on professional audiences, future efforts must expand to systematically include patients in shaping how AI fits into care processes and SDM. Without active efforts to co-design and involve patients in development and to train clinicians in communicating about AI, there is a risk of eroding trust or creating asymmetries in SDM. Thus, the findings reinforce that effective integration of AI into SDM is not only a technical challenge but also a collaborative and communicative one. For AI to genuinely promote SDM in transplant care, it must be introduced in a way that strengthens, rather than displaces, the patient–support person–physician relationship.

Although participants referred to “AI” in AI-based DSS as a general concept, it is important to note that contemporary medical AI applications encompass a wide range of modalities with distinct implications for trust, transparency, and decision-making. These may include predictive machine learning used for risk stratification or outcome prediction, as well as conversational agents or generative AI applications that directly interact with end users. Such modalities differ substantially in their visibility, interaction style, explainability, and user design. While “background” predictive AI models may be experienced as less intrusive, interactive AI systems may be perceived as more present in the medical encounter and thus more directly affect the patient-physician relationship [46]. Interpreting participants’ concerns and expectations through this lens helps to contextualize why issues such as the preservation of the “human touch,” accountability, and transparency were particularly noticeable in our findings.

### The “Human Touch” in the Age of AI: Empathy, Emotion, and Patient Perspectives

Interestingly, while many participants doubted that AI could replicate the so-called “human touch,” this skepticism merits deeper examination. The term “human touch” was used by participants to denote a complex interplay of empathy, emotional understanding, nonverbal cues, and situational sensitivity—aspects that are perceived as central to patient-centered care. This translates into the ability to recognize and respond to a patient’s emotional state, convey reassurance, and build rapport—capacities typically associated with human caregivers. According to most of the participants (26/36, 73%), the concept of empathy is presumed to be uniquely human. However, emerging studies suggest that AI-based chatbots or support tools may sometimes be perceived as more empathetic or nonjudgmental than rushed or fatigued physicians, especially in structured communication contexts (eg, symptom checks, emotional support) [8,47-49]. While our participants viewed AI as inherently lacking empathy, it remains unclear whether this is an ontological limitation or simply a function of current design and communication strategies. Across interviews, a recurring pattern suggested differences between patients and support persons in how AI modalities were evaluated. While not uniformly expressed by all participants, support persons more frequently emphasized the practical benefits of predictive, background AI systems that support physicians in risk monitoring and data processing. In contrast, patients more often articulated ambivalence when AI was described as directly interacting with them or shaping physician-patient communication. While some support persons appeared more receptive to background predictive AI systems, and several patients expressed ambivalence toward more interactive or conversational AI, these patterns were not uniformly observed across all interviews. Rather than representing a consistent role-based distinction, these differences should be understood as interpretive tendencies within the data, shaped by individual experiences, expectations, and levels of engagement with AI. These patterns suggest that AI tools may need to address the distinct informational and emotional needs of patients and their support persons. However, this area warrants further investigation as the perceived emotional sufficiency of AI may shape both trust and acceptance of end users.

### AI Literacy and the “AI Package Leaflet”: Building Understanding and Trust Through Transparent Information

The findings highlight that knowledge gaps are a significant barrier to the acceptance of AI in SDM. Patients and support persons often expressed uncertainty about how AI arrives at its recommendations, who controls the data, and what exactly the system “knows” about them. To address this, structured informational tools, such as package leaflets for AI systems, may help bridge this gap [28,34,50]. Such a tool might include: The purpose of the AI-DSS, what type of data it uses, how it generates risk estimates, what its limitations are, how well it performs for specific subgroups, and a brief disclaimer of who is accountable for final decisions. This concept aligns with emerging international efforts to enhance algorithmic transparency and patient-centered communication [44,45]. In this light, an “AI package leaflet” could serve as a translational interface between technical system design and patient-centered AI-based care and decision-making. It would also support the ethical imperative of informed consent, which in the context of AI not only requires understanding treatment options but also the nature and role of digital tools involved [51]. Ideally, such information should also be co-designed with patients, evaluated for health literacy, and tested in different clinical settings to ensure it meets the diverse informational needs of users [51]. As an illustrative example (not derived from participant data), an “AI package leaflet” could briefly outline what the system does, what type of data it uses, how recommendations are generated, and what its limitations are. Transparent, accessible information like this is key to supporting informed consent, fostering trust, and empowering patients to participate actively in AI-assisted SDM. AI literacy not only shapes general attitudes but also affects how patients and support persons participate in SDM during consultations. If patients do not understand how an AI-based risk estimate is generated, they may feel unable to question or discuss it meaningfully. In contrast, basic understanding of the system’s purpose and limitations enables more active engagement, supporting genuinely collaborative decision-making. AI literacy emerged as a key factor shaping how patients engaged with AI-DSS, particularly within SDM processes. In practical terms, limited understanding of AI systems may constrain patients’ ability to question recommendations, interpret uncertainty, or actively participate in deliberation. Within clinical workflows, this suggests that AI outputs must not only be explained but also contextualized within the decision-making process, enabling patients to understand how recommendations relate to their individual situation, preferences, and alternatives. Without such integration, AI risks reinforcing passive acceptance rather than supporting meaningful SDM.

### Limitations

Limitations of this study may include potential social desirability and interviewer effects. Given the sensitive nature of discussing personal health decisions and emerging technologies such as AI, participants may have been inclined to express favorable views or align their responses with what they perceived to be the interviewer’s expectations. In addition, participants’ baseline familiarity with AI or digital health tools was not systematically assessed. As a result, differences in technological literacy may have influenced how participants understood and reflected on the role of AI in SDM processes. The study is subject to recall bias as participants reflected retrospectively on clinical appointments. The sample was recruited purposefully at a single German transplant center, which limits the generalizability of the findings to broader transplant populations.

### Conclusions

Our findings support previous research suggesting that the needs and expectations of patients regarding AI technologies have often not been explicitly studied [9,10]. In the context of nephrology and SDM, our study shows that patients and their support persons particularly emphasize concerns about a potential loss of the “human touch” in treatment due to increased reliance on AI, as well as a lack of trust in AI-based DSS stemming from limited understanding of these technologies [31,52].

However, participants welcomed AI-based DSS as a supportive tool and simultaneously emphasized its role as a supplement in SDM—not a replacement for human judgment. Furthermore, the successful integration of AI-based DSS into posttransplant care may depend less on technical precision and more on its alignment with the interpersonal and ethical foundations of SDM, including the preservation of the “human touch.”

Uncertainty about how AI works, its potential benefits, and its challenges for medical encounters revealed patients’ knowledge gaps, highlighting the need for further patient involvement in the design of these tools as well as improved patient information strategies, such as AI “package leaflets.” Such strategies may strengthen patient trust through improved patient-physician communication on AI and help patients and support persons become involved in decisions related to their care. Future research should explicitly differentiate between AI modalities when examining patients’ perceptions, acceptance, and experiences with AI-based DSS. Comparative studies are needed to assess how predictive background AI-based systems versus interactive AI applications are perceived and integrated into decision-making processes by patients and their support persons.

## Abbreviations

AI: artificial intelligence
CONSORT-AI: Consolidated Standards of Reporting Trials—Artificial Intelligence
DSS: decision support system
KTC: kidney transplant center
SDM: shared decision-making
SPIRIT-AI: Standard Protocol Items: Recommendations for Interventional Trials—Artificial Intelligence
